# Large-Language Models in Orthodontics: Assessing Reliability and Validity of ChatGPT in Pretreatment Patient Education

**DOI:** 10.7759/cureus.68085

**Published:** 2024-08-29

**Authors:** Stratos Vassis, Harriet Powell, Emma Petersen, Asta Barkmann, Beatrice Noeldeke, Kasper D Kristensen, Peter Stoustrup

**Affiliations:** 1 Section of Orthodontics, Department of Dentistry and Oral Health, Aarhus University, Aarhus, DNK; 2 Section of Orthodontics, Department of Dentistry and Oral Health, Aarhus Universiy, Aarhus, DNK; 3 Department of Dentistry and Oral Health, Aarhus University, Aarhus, DNK; 4 Department of Oral and Maxillofacial Surgery, Aarhus University Hospital, Aarhus, DNK

**Keywords:** artificial intelligence in dentistry, ai orthodontics, digital orthodontics, patient education, large language models (llm)

## Abstract

Background: Patients seeking orthodontic treatment may use large language models (LLMs) such as Chat-GPT for self-education, thereby impacting their decision-making process. This study assesses the reliability and validity of Chat-GPT prompts aimed at informing patients about orthodontic side effects and examines patients' perceptions of this information.

Materials and methods: To assess reliability, n = 28 individuals were asked to generate information from GPT-3.5 and Generative Pretrained Transformer 4 (GPT-4) about side effects related to orthodontic treatment using both self-formulated and standardized prompts. Three experts evaluated the content generated based on these prompts regarding its validity. We asked a cohort of 46 orthodontic patients about their perceptions after reading an AI-generated information text about orthodontic side effects and compared it with the standard text from the postgraduate orthodontic program at Aarhus University.

Results: Although the GPT-generated answers mentioned several relevant side effects, the replies were diverse. The experts rated the AI-generated content generally as “neither deficient nor satisfactory,” with GPT-4 achieving higher scores than GPT-3.5. The patients perceived the GPT-generated information as more useful and more comprehensive and experienced less nervousness when reading the GPT-generated information. Nearly 80% of patients preferred the AI-generated information over the standard text.

Conclusions: Although patients generally prefer AI-generated information regarding the side effects of orthodontic treatment, the tested prompts fall short of providing thoroughly satisfactory and high-quality education to patients.

## Introduction

Artificial intelligence (AI) approaches are gaining increased attention to support clinicians in the field of orthodontics [1]. Large language models (LLM) such as Generative Pretrained Transformer 4 (GPT-4) have been developed with cognitive capabilities to aid users in a wide array of tasks. LLM functions by analyzing vast amounts of text data to learn language patterns and subsequently to generate responses based on this learned data, using statistical probabilities to create coherent and contextually relevant text. Prompts for LLMs, manifesting as inquiries [2], are adaptable to different languages and can encompass various types of inputs, such as spreadsheets, research papers, or code. GPT has additionally been encoded with medical knowledge that can be utilized in clinical settings by patients to address their health-related concerns and patient education [3]. In the realm of orthodontics, this implies that patients seeking to initiate orthodontic treatment may opt to utilize LLM platforms for self-education. As the utilization of human-computer interaction through LLMs for patient education and consultation becomes increasingly popular, certain risks will arise. For example, false responses, referred to as "hallucinations" [4], could potentially influence patients in their decision-making process. Hence, it is essential to validate the outputs generated by LLMs such as ChatGPT to ensure their reliability and validity [5].

Aims and objectives

Expanding the extensive body of literature reviews addressing the intersection of AI and orthodontics [6-9], this study aims to evaluate the ability of GPT to generate educational information for orthodontic patients based on quantitative data. More specifically, the study aims to investigate: (1) How does individualized and standardized prompting affect the reliability of the GPT output? (2) How well does GPT provide information on orthodontic side effects based on expert evaluation (content validity)? (3) How do patients’ attitudes and perceptions differ between AI-generated information about orthodontic side effects compared with the standardized conventional informational form provided by the Section of Orthodontics at Aarhus University, Denmark (clinical face validity)?

## Materials and methods

The study was conducted at the Section of Orthodontics, Department of Dentistry and Oral Health, from September 2023 to January 2024. It was conducted in accordance with the regulations of the Danish Health Authorities regarding questionnaire studies. All included subjects gave consent to participate in the present study.

Reliability and content validity of ChatGPT responses

The first part of the study aimed to assess the reliability and content validity of ChatGPT responses based on standardized and individualized prompts. We invited 28 subjects to participate in this part of the study. The randomly selected subjects were recruited from the patients who presented themselves at the University of Aarhus, Department of Oral Health and Dentistry. Inclusion criteria were that the subjects were not undergoing orthodontic treatment nor did so in the past because we wanted this group of participants to be naïve to information about orthodontic treatment. Patients who already received information related to orthodontic treatment and those without access to ChatGPT were excluded from the study. The included subjects were asked to log into their ChatGPT account (Oct 23 Version; OpenAI; https://chat.openai.com/). Fourteen randomly selected participants were asked to use the free-of-charge ChatGPT-3.5 version, and the other 14 participants were asked to use the ChatGPT-4.0 version, which requires payment. Initially, the subjects were asked to generate information from ChatGPT about side effects related to orthodontic treatment using self-formulated prompts. Second, the subjects were asked to generate ChatGPT responses about side effects related to orthodontic treatment using a standardized prompt: “What are the side effects of orthodontic treatment?”. A screenshot of the outputs was generated. The content of the outputs was categorized in tables.

Three experts with special knowledge about side effects related to orthodontic treatment evaluated the overall content validity (“quality of information provided”) of the ChatGPT-generated responses to the self-formulated prompts and the standardized prompt. The expert team consisted of two certified orthodontists with more than ten years of work experience each and one postgraduate student. The evaluators individually assessed the ChatGPT responses using a five-point Likert scale (0=Completely deficient, 1=Partially deficient, 2=Neither/nor, 3=Partially satisfactory, 4=Completely satisfactory). The raters evaluated the generated answers separately for ChatGPT-3.5 and ChatGPT-4 as well as self-formulated vs. standardized prompts.

Clinical face validity of ChatGPT responses

The second part of the study aimed to assess patients’ attitudes and perceptions toward AI-generated information about orthodontic effects. We invited all consecutive orthodontic patients to participate in this part of the study. Patients under the age of 13 were excluded from the study. Inclusion criteria included currently undergoing treatment or post-treatment retention visits at the Section of Orthodontics, Department of Dentistry and Oral Health, Aarhus University. The enrolled patients were initially presented with two documents informing them about side effects related to orthodontic treatment. The first document was the sectional standard document for patient information and consent. The second document was a ChatGPT-generated description of side effects from orthodontic treatment using the standardized prompt: “What are the side effects of orthodontic treatment?”. Subsequently, a quantitative questionnaire was employed on the enrolled patients to gauge their attitudes and perceptions of the presentation of the orthodontic side effects. The patients were asked to rate the content of the two documents on a five-point Likert scale for the following aspects: (1) “Usefulness of the content” (scale 0-5, 0="not useful at all," 5="very useful”), (2) “Comprehensibility of the content” (scale 0-5, 0="not understandable at all," 5="Very easy to understand," (3) "How anxious/afraid does the content make you about experiencing side effects?” (Scale 0-5, 0="Very anxious/afraid," 5="not anxious/afraid at all"). Finally, the patients were asked to indicate which of the two documents they preferred.

Statistics

Descriptive statistics was used to report the results of the data, including the relative occurrence of individual side effects in ChatGPT responses (for reliability testing) and providing mean and standard deviations for presenting data related to content validity and clinical face validity. Wilcoxon Ranksum tests were used to assess inter-group differences in the assessment of content validity and clinical face validity. We calculated the interrater class coefficient (ICC) to indicate agreement between the experts.

## Results

Reliability of ChatGPT responses

**Figure 1:**
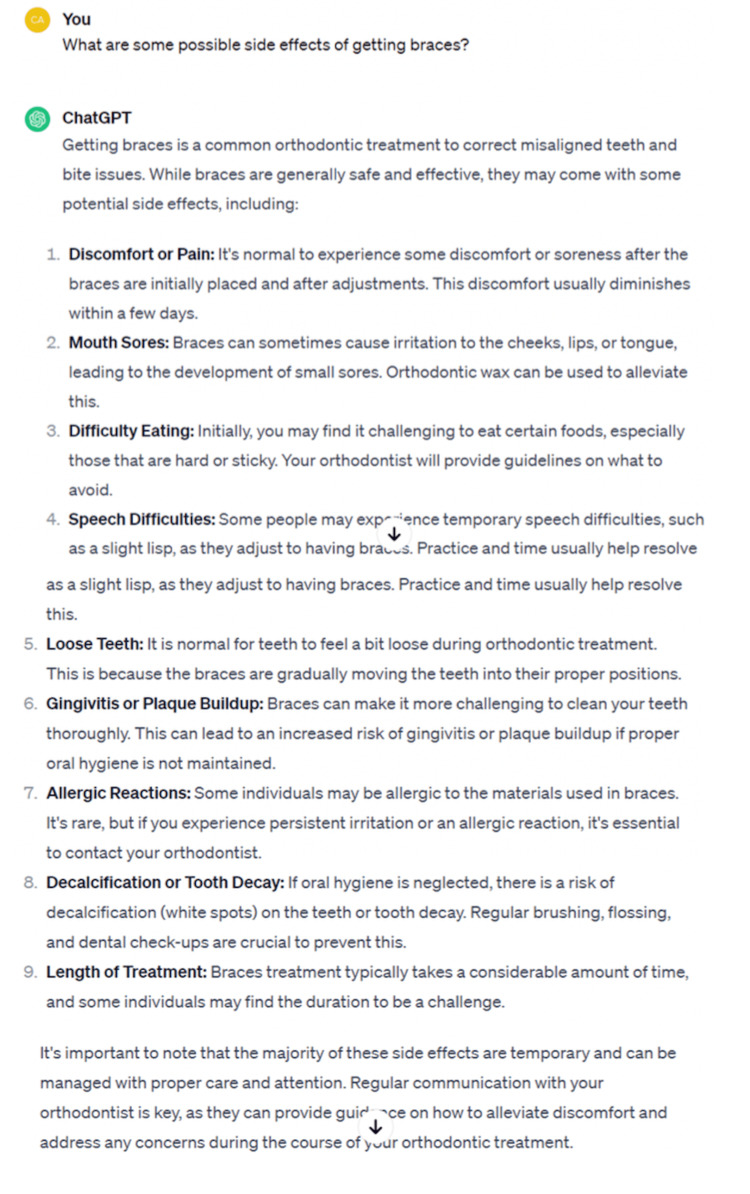
Example for prompt and reply in ChatGPT-3.5.

**Figure 2:**
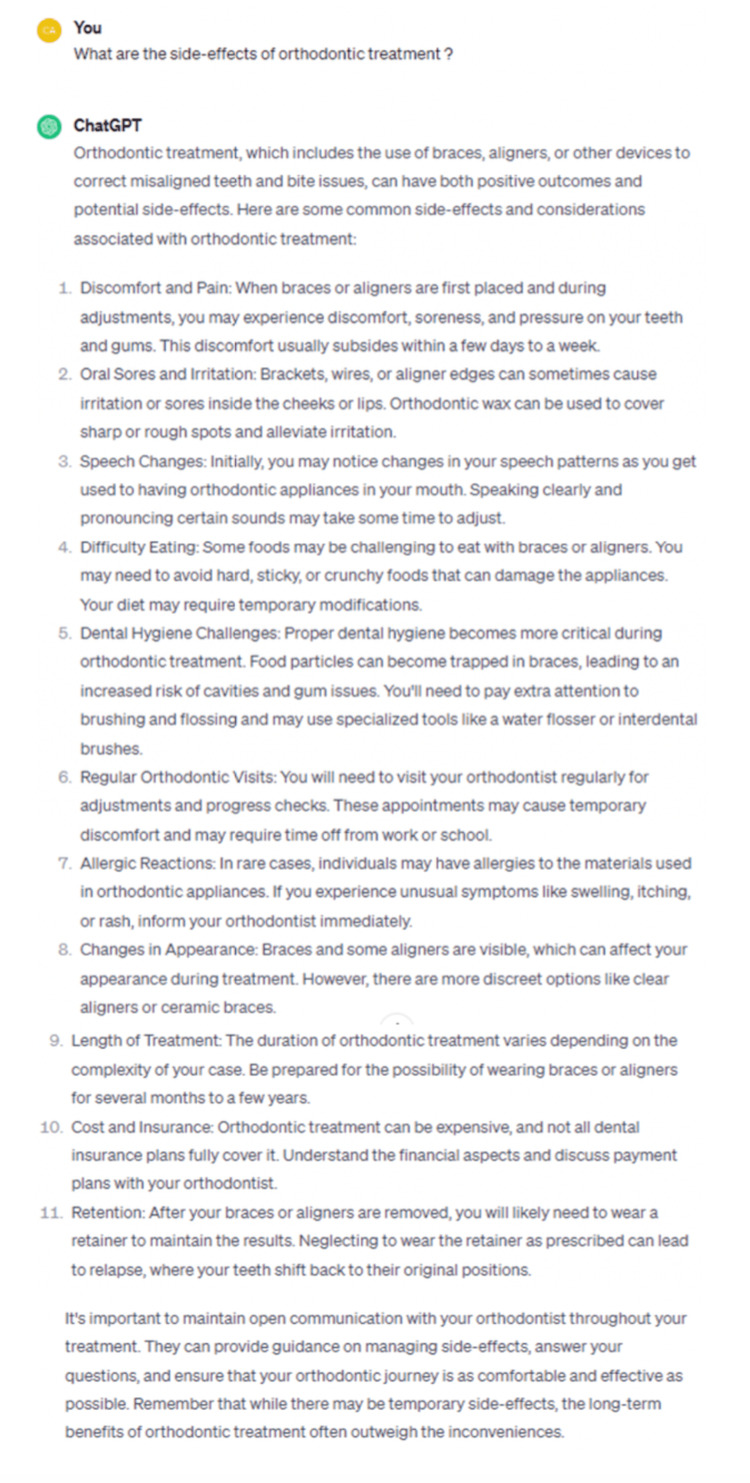
Example for prompt and reply in ChatGPT-4.

We included 28 participants for this part of the study with a mean age of 32.2 years (SD=12.9). The chatbot responses to the self-formulated and standardized prompts are listed in Table 1. Figures 1-2 provide examples of the prompts and answers for ChatGPT-3.5 and ChatGPT-4. On average, the self-formulated prompts mentioned 6.7 different side effects for ChatGPT-3.5 (SD=2.5) and 7.3 for ChatGPT-4 (SD=3.3). The standardized prompts lead to a higher number of side effects mentioned, on average 7.8 for ChatGPT-3.5 (SD=2.0) and 9.2 for ChatGPT-4 (SD=2.6). Although some side effects were mentioned in nearly all replies, over 30 were mentioned overall, with 14 side effects (such as bone loss or ankylosis) only being mentioned in 4 or fewer answers, indicating diversity in the generated replies. Table 2 displays the list of the self-formulated prompts.

**Table 1:** Side effects were mentioned in the LLM-generated replies. The table displays the ten most common side effects generated by GPTs. *One reply for ChatGPT-4 was missing.

		GPT-3.5 (n=28)		GPT-4* (n=27)		Combined (GPT-3.5 and GPT-4*)	
Side effects mentioned (Top 10)	Total number of mentions	Self-formulated prompts	Standardized prompts	Self-formulated prompts	Standardized prompts	Self-formulated prompts	Standardized prompts
Pain and discomfort	53	14	14	12	13	26(93%)	27(100%)
Speech changes/difficulties	45	11	14	8	12	19(68%)	26(96%)
Oral irritations and soreness	44	12	12	10	9	22(79%)	22(81%)
Difficulties eating	32	7	10	6	9	13(46%)	19(70%)
Allergic reactions	32	5	9	8	10	13(46%)	19(70%)
Dental oral hygiene challenges	31	8	8	8	7	16(57%)	15(56%)
Gingivitis or gingival damages	26	7	6	4	9	11(39%)	15(56%)
Root resorption	24	2	4	8	10	10(36%)	14(52%)
Decalcification and tooth decay	18	2	1	8	7	10(36%)	8(30%)
Gum and jaw pain or TMJ issues	14	2	1	4	7	6(21%)	8(30%)

**Table 2:** Self-formulized prompts. Note: To be compared to standardized prompt "What are the side-effects of orthodontic treatment?".

Self-formulized prompts
I'm getting brackets soon. Can you tell me about the potential side effects of getting brackets?
What are the possible side effects of a possible treatment with brackets?
Hey! My kid needs brackets, and I'm wondering, are there any serious side effects or potential harm from braces?
What are the side effects of orthodontics?
I'm interested in knowing the possible side effects of getting brackets. Can you help me? I don't feel like I got enough information from the brochure my dentist gave me.
Hello! What are the possible side effects and disadvantages of getting brackets?
Possible side-effects of a dental treatment with brackets for a 43-year-old male.
I have a friend that is getting a dental treatment with brackets and is a little nervous of the side effects. Can you list some possible side-effects or concerns that arises from getting this dental treatment?
What are the consequences of dental brackets?
Hey, do you know about brackets? I didn't get them as a kid, and now I’m worried it's going to hurt a lot since I am starting so late (I'm 28) so that probably means that things aren't as flexible anymore. They also said they need to two teeth to make room; won't I just end up with giant empty gaps when the teeth move back later?
What should I be afraid of if I have to have my teeth straightened?
I am going to get dental brackets soon. Can you tell me something about the potential side effects of wearing brackets that I should be aware of?
I'm thinking about getting dental brackets, should I be worried about the side-effects with the treatment?
Side effects of orthodontic treatment?
I am to get brackets. What potential side-effects should I be aware of before getting the treatment?
I would like to know what the side effects are of getting brackets?
Hi. I'm considering having brackets, but I am concerned about possible side effects. What are the side effects and is it worth having brackets despite the risk?
Explain the side-effects of having brackets in your mouth
What are the side effects of having teeth straightened?
What are some possible side effects of getting brackets?
I would like to know what the side effects of dental brackets may be, especially whether there will be any pain.
Do you know what the possible side-effects are of dental treatment with brackets?
Are there side effects of getting brackets bonded at the dentist?
What side effects can I expect if I get brackets on my teeth?
I have front teeth that have crowding. And I would like to wear brackets and have my teeth straightened. Are there any side effects to that treatment? I am about to get brackets on the teeth. What should I be nervous about?
Side effect of dental treatment.
What kind of side effects can I expect to observe throughout the duration of a dental treatment with brackets

Content validity of ChatGPT responses

**Figure 3:**
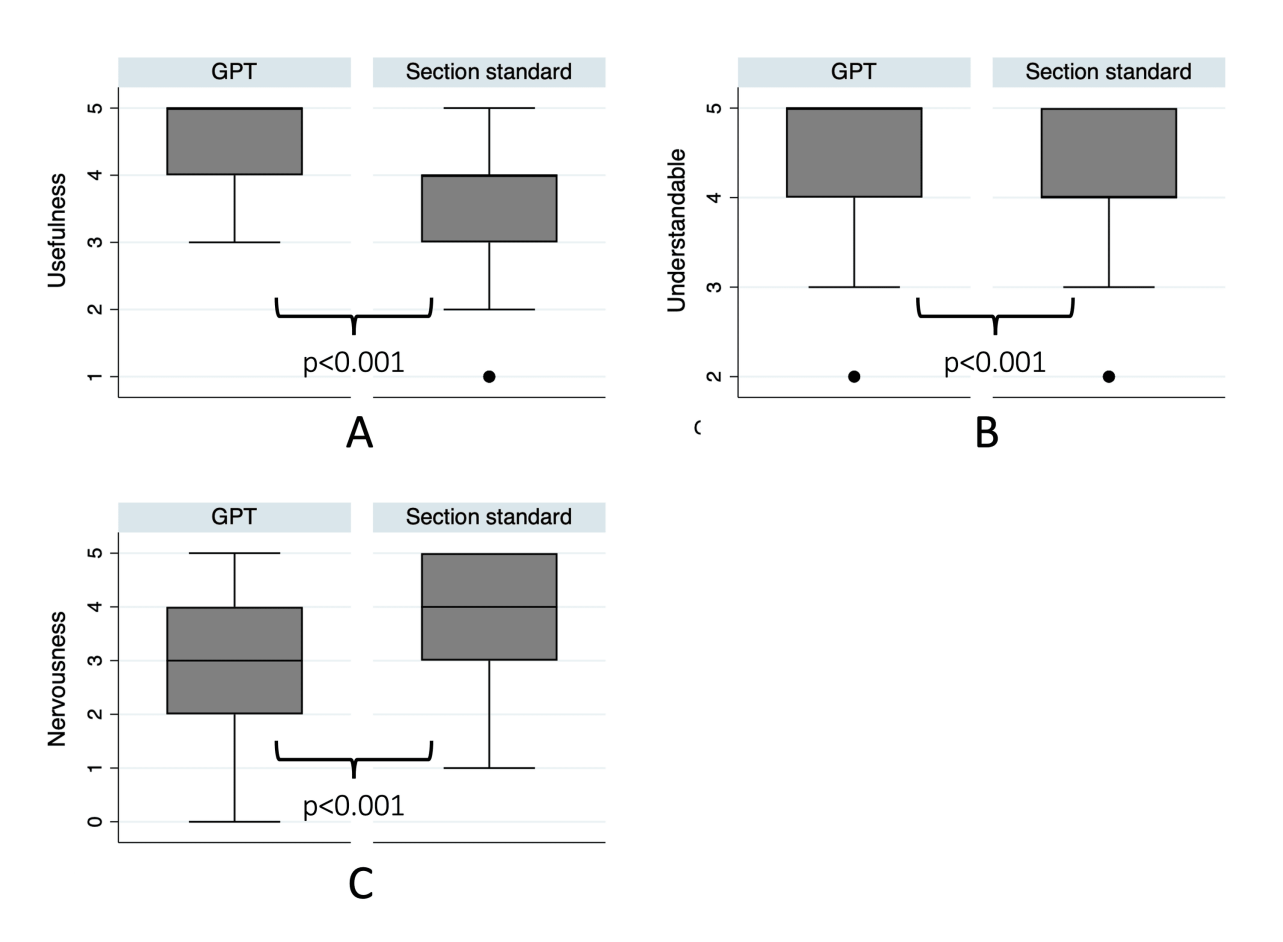
Patient perceptions of LLM generated side effect explanations. Note: Wilcoxon ranksum test. (a) Patient perception of text usefulness. Measured on a six-point Likert scale: 0=“not useful at all,” 5=“very useful.” (b) Patient perception of text comprehensibility. Measured on a six-point Likert scale, 0=“not understandable at all,” 5=“very easy to understand.” (c) Patients’ perceived anxiousness when reading the text. Measured on a six-point Likert scale: 0=”very little anxious/afraid,” 5=”very much anxious/afraid.

**Figure 4:**
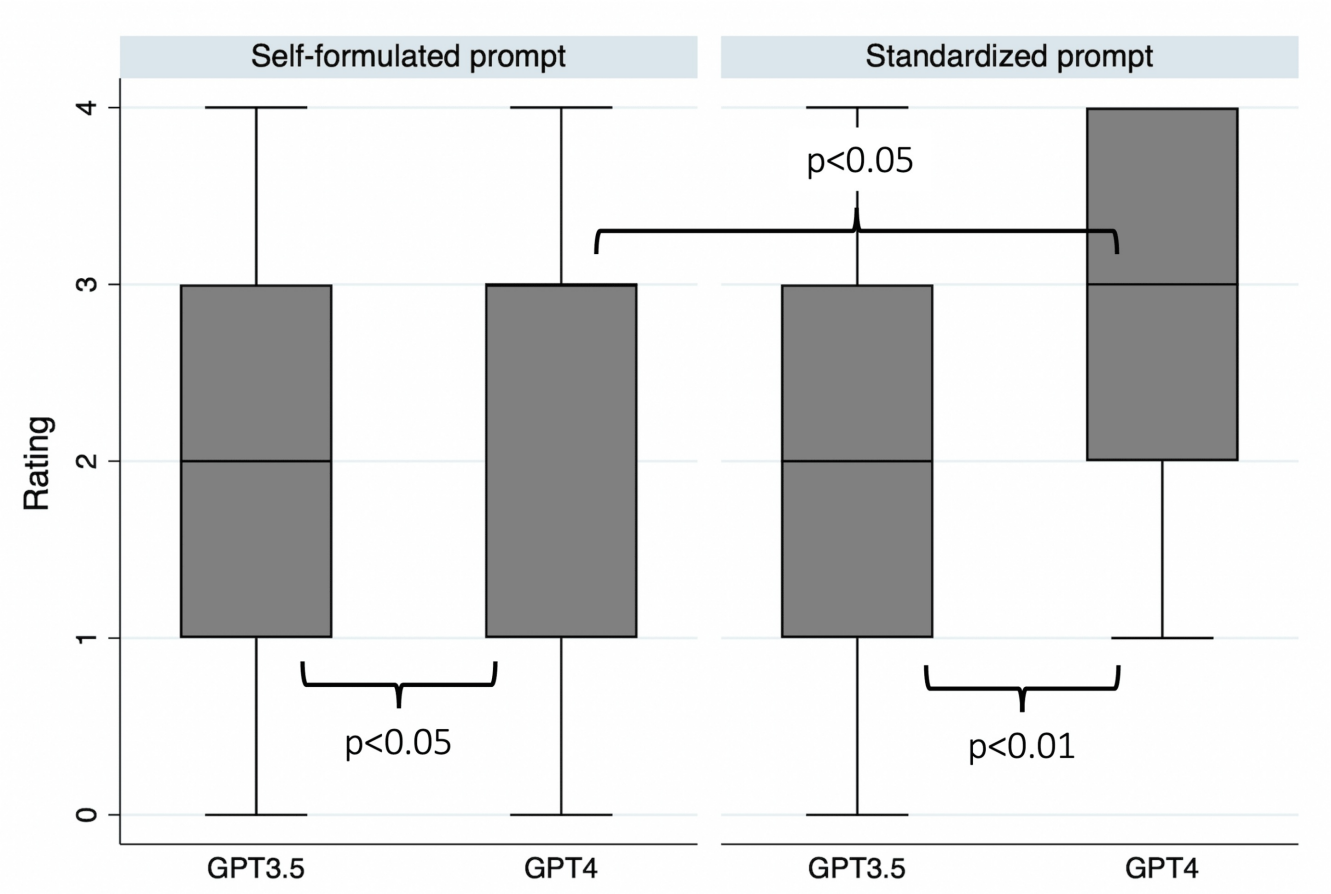
Content validity of self-formulated and standardized prompts assessed by orthodontic experts. Note: Measured on five-point Likert-scale: 0=Completely deficient, 1=Partially deficient, 2=Neither/nor, 3=Partially satisfactory, 4=Completely satisfactory. Wilcoxon Ranksum test.

The mean outcome of the experts’ quality assessment of the GPT responses is displayed in Figure 3. Overall, the content validity of the GPT-4 answers was rated significantly higher than the GPT-3.5 answers (p<0.001). The experts also rated the answers to the standardized prompts significantly higher than the self-formulated prompts (p<0.05). More specifically, the raters evaluated the self-formulated prompts with an average of 1.69 (SD=1.05) for GPT-3.5 and 2.31 (SD=1.20) for GPT-4, indicating “neither deficiencies nor satisfaction” (p<0.05) (Figure 4). For the standardized prompts, the raters rated the GPT-3.5 answers with an average of 2.05 (SD=1.15), indicating “neither deficiencies nor satisfaction,” and the standardized prompts generated using GPT-4 achieved the highest score of 2.90 (SD=1.05) from the experts, indicating “partial satisfaction” (p<0.01). Within GPT-4, the standardized prompts received significantly better ratings than the self-formulated prompts (p<0.05).

The inter-rater agreement was very high. For self-formulated prompts, the ICC among the three raters was 90.93 (95%CI: 0.78; 0.97) for ChatGPT-3.5 and 94.75 (95%CI: 0.87; 0.98) for ChatGPT-4. For standardized prompts, the ICC was 0.93 (95%CI: 0.84; 0.98) for ChatGPT-3.5 and 0.88 (95%CI: 0.68; 0.96) for ChatGPT-4, indicating very high agreement among the raters for all categories.

Clinical face validity of ChatGPT responses

A cohort of 46 orthodontic patients completed the questionnaire about the two documents. Table 3 displays background information on the sample.

**Table 3:** Background information of study cohort consisting of orthodontic patients.

Variable	
Median age (25^th^ and 75^th^ percentiles)	21 years (15–29 years)
Sex (proportion)	
Female	35 (76.1%)
Male	11 (23.9%)
Where in treatment	
Pre-treatment	5 (10.9%)
In active treatment	28 (60.9%)
In retention	11 (23.9%)
Other	2 (4.4%)
Types of treatment	
Before treatment	1 (2.2%)
Fixed appliance	36 (78.3%)
Aligner	3 (6.5%)
Functional appliance	2 (4.3%)
Other	4 (8.7%)

Output usefulness was rated 4.46 (SD=0.72) for the GPT-generated text and, therefore, significantly higher than the section’s text, which had an average rating of 3.74 (SD=1.00) (p<0.001). Similarly, understandability was higher for the GPT-generated text, with an average of 4.63 (SD=0.71) compared to the average of 4.13 (SD=0.81) for the section’s text (p<0.001). Lastly, the section’s text made the patients significantly more nervous, with an average of 3.70 (SD=1.09) compared with the GPT-generated text average of 3.11 (SD=1.55) (p<0.001). Overall, 78.3% of the respondents preferred the GPT-generated text over the standard information. Only 15.2% preferred the standard information sheet, and 6.5% were indifferent. More detailed information is presented in the supplementary material section (Appendix).

## Discussion

The rapid development in artificial intelligence and LLMs indicates a future potential for self-education of orthodontic patients. This is, to the best of our knowledge, the first study to assess the reliability and validity of LLM responses for pre-treatment patient education in orthodontics, and our findings show that: (1) There is a moderate variation in chatbot responses to GPT prompts. (2) The quality (content validity) of the GPT answers was of moderate quality and significantly depended on the way the prompts are posed and the type of chatbot used (GPT-3.5 vs. GPT-4). (3) From the patients' and users' perspective, the GPT chatbot's output is deemed useful and comprehensible without eliciting substantial fears about potential side effects. This user-friendly perception is believed to play a key factor in most users' preference for GPT-based information over the standard information given at the university before initiating treatment.

The reliability analysis shows that certain side effects like “pain and discomfort” were mentioned in nearly all of the artificially generated explanations, showing a high degree of reliability. The top ten list of the most mentioned side effects includes the most common adverse effects, namely root resorption, pain, periodontal disease, pulp vitality, and temporomandibular disorders [10]. However, the high number of total side effects mentioned in all the replies and the high number of side effects only mentioned in a few replies show the diversity in the GPTs' replies. Even for the standardized prompts, GPT provides a range of different replies, demonstrating that GPT is not consistent and, therefore, not reliable in listing all relevant side effects. It should be noted that information about root resorption and the risk of developing demineralization was only mentioned in 30-52% of the responses from GPT. These risks are considered essential information for informed consent before orthodontic treatment. The occasional lack of comprehensive information from GPT reflects the complex and non-deterministic nature of LLMs and highlights a challenge in relying on them for complete patient education. The slightly lower number of side effects mentioned for the self-formulated prompts indicates that knowledge about creating adequate prompts might impact the reply quality [11]. The side effects mentioned relate mostly to fully fixed appliances but do not specifically consider aligners or removable and lingual appliances.

Although the experts rated the content validity of some of the GPT answers as "satisfactory," the AI-generated results, on average, were neither satisfactory nor deficient, indicating a lack of quality to fully inform the patients about orthodontic side effects. Hallucinations and genuine misinformation were not discovered in the responses retrieved. The fact that the GPT-4 answers achieved a significantly higher score from the experts might hint at the future potential of GPT for informing patients. As the standardized prompts achieved a higher score from the experts, the results might improve as the patients become more familiar with prompting and enhance their respective skills in the future or use several prompts [12].

In contrast to the experts’ evaluation, the patients expressed satisfaction with AI-generated information about orthodontic side effects with respect to the comprehensibility, usefulness, and psychological impact of the text. However, clinicians face legal and ethical concerns when they present wrong or too little information to their patients, in contrast to LLMs [13], which might complicate the information presentation.

Another critical consideration is that LLM outputs depend on the input provided by humans; e.g., it fully depends on the quality of the provided prompts [14]. Hence, if a patient does not provide all relevant data or fails to communicate clearly, decision-making can be negatively impacted as AI might fail to consider and investigate further factors relevant to individualized treatment [15]. 

Despite these concerns, LLMs appear to have the potential to assist orthodontic patient education by providing quality information about orthodontic conditions, which might be difficult to obtain elsewhere. It is also conceivable that specialized orthodontic chatbots based on LLMs could make their way into the commercial market. Overall, there seems to be a potential place for AI-generated patient education to supplement the statutory information provided by the doctor prior to the start of any orthodontic treatment.

Several limitations should be noted. Orthodontic side effects, as the topic chosen for investigation, is a broad topic. It is unclear to what extent previous conversations with the Chatbot influenced the answers to the investigated prompts, as we do not know whether the prompt has started a new conversation. Also, previous knowledge about prompting and creating several prompts can impact the quality of GPT's replies to self-formulized prompts. In general, LLMs can face various limitations. For example, results generated from LLMs can be subject to bias if the information used to train the model was already biased. Besides, LLMs can in certain cases present wrong information due to information hallucination or reasoning errors [11]. Future research could investigate to what extent LLMs can comply with legal requirements when informing about side effects and used to generate treatment consent forms. ChatGPT 3.5 and 4 are just two of several commercial LLM models that are readily available. Future studies should also examine the quality of other LLMs (e.g., Google Bard and Microsoft Bing), as research has shown significant variation in the quality of output when different LLM models are utilized [16].

## Conclusions

LLMs like GPT can interfere with patient-orthodontist interactions. Although patients generally prefer AI-generated information regarding orthodontic treatment side effects, the tested prompts do not provide thoroughly satisfactory education of high quality to patients, indicating that AI-generated information as tested here is not able to replace a consultation at the orthodontic clinic to fully inform the patients.

## Appendices

**Table 4:** Comparison of information sources.

Variable	Outcomes	Standard information (median and 25^th^; 75^th^ percentiles)	ChatGPT responses (median and 25^th^; 75^th^ percentiles)	p-value*
How useful was the information?	0 = very little; 5= = really useful	4 (3;4)	5 (4;5)	0.0002
How understandable was the information?	0 = very difficult; 5 = very easy	4 (4;5)	5 (4;5)	0.0005
Did the information make you nervous about getting the side effects?	0 = very little; 5 = very much	4 (3;5)	3 (2;4)	0.000
Which of the two information texts do you prefer?	N (proportion)	7 (15.2%)**	36 (78.3%)**	-
